# Spatial distribution of violence rates against pregnant women in Brazil in 2023

**DOI:** 10.15649/cuidarte.5584

**Published:** 2026-08-21

**Authors:** Rayssa Milena Palasi Semezatto, Maria Luiza Melo da Silva, Lays Silva de Azevedo, Emiliana Cristina Melo, Rosana Rosseto de Oliveira

**Affiliations:** 1 Centro Universitário Ingá-Uningá, Maringá, Brazil. E-mail: milenasemezatto123@gmail.com Centro Universitário Ingá-Uningá Maringá Brazil milenasemezatto123@gmail.com; 2 Universidade Estadual de Maringá - UEM, Maringá, Brazil. E-mail: luizamah543@gmail.com Universidade Estadual de Maringá Maringá Brazil luizamah543@gmail.com; 3 Universidade de São Paulo, São Paulo, Brazil. E-mail: laysaz@outlook.com Universidade de São Paulo São Paulo Brazil laysaz@outlook.com; 4 Universidade Estadual do Norte do Paraná, Bandeirantes, Brazil. E-mail: ecmelo@uenp.edu.br Universidade Estadual do Norte do Paraná Bandeirantes Brazil ecmelo@uenp.edu.br; 5 Centro Universitário Ingá-Uningá, Maringá, Brazil. E-mail: rosanarosseto@gmail.com Centro Universitário Ingá-Uningá Maringá Brazil rosanarosseto@gmail.com

**Keywords:** Epidemiology, Pregnant Women, Notification, Women's Health, Violence, Epidemiología, Mujeres Embarazadas, Notificación, Salud de la Mujer, Violencia, Epidemiologia, Gestantes, Notificação, Saúde da Mulher, Violência

## Abstract

**Introduction:**

Violence against pregnant women is a multifactorial public health problem that can worsen maternal and fetal health, making its analysis relevant for understanding the phenomenon.

**Objective:**

Describe the spatial distribution of notification rates of violence against pregnant women in Brazil.

**Materials and Methods:**

This was a cross-sectional ecological study of notification rates of violence against pregnant women residing in Brazil in 2023, obtained from the Notifiable Diseases Information System, with data extracted in March 2025. The spatial distribution of notification rates of violence against pregnant women was analyzed according to socioeconomic indicators (race/skin color, age group, and educational level). Global and Local Moran’s Indexes were used for spatial analysis, considering immediate regions as units of analysis.

**Results:**

The notification rate of violence against pregnant women in Brazil was 7.82 per 1,000 live births (19,851 notifications). The states of Amazonas, Roraima, Acre, Mato Grosso do Sul, and Paraná stood out with high rates. Higher occurrence was observed among women of mixed-race (brown), Asian, and Indigenous race/skin color, as well as among those under 20 years of age and with low educational attainment.

**Discussion:**

Brazil is marked by regional disparities, which are reflected in indicators of violence against pregnant women.

**Conclusion:**

Violence against pregnant women is present throughout the country, affecting all race/skin color groups, age groups, and educational levels. However, variations and specificities among regions were identified. Thus, the data obtained represent a relevant source of information and may support the planning and implementation of interventions.

## Resumen

**Introducción:**

La violencia contra gestantes es un problema de salud pública multifactorial que puede agravar la salud materna y fetal, siendo relevante su análisis para la comprensión del fenómeno.

**Objetivo:**

Describir la distribución espacial de las tasas de notificaciones de violencia contra gestantes en Brasil.

**Materiales y métodos:**

Estudio ecológico de corte transversal de las tasas de notificación de violencia contra mujeres embarazadas residentes en Brasil ocurridas en 2023, obtenidas a partir del Sistema de Información de Enfermedades de Notificación, con datos extraídos en marzo de 2025. Se realizó la distribución espacial de las tasas de notificación de violencia contra mujeres embarazadas según indicadores socioeconómicos (raza/color de piel, grupo etario y nivel educativo). Para el análisis espacial se emplearon los índices de Moran Global y Local, considerando las regiones inmediatas como unidades de análisis.

**Resultados:**

La tasa de notificación de violencia contra mujeres embarazadas en Brasil fue de 7,82 por cada 1.000 nacidos vivos (19.851 notificaciones). Los estados de Amazonas, Roraima, Acre, Mato Grosso do Sul y Paraná se destacaron por presentar tasas elevadas. Se observó mayor ocurrencia en mujeres de raza/color de piel parda, amarilla e indígena, así como en aquellas menores de 20 años y con bajo nivel educativo.

**Discusión:**

Brasil está marcado por disparidades regionales, las cuales se reflejan en los indicadores de violencia contra mujeres embarazadas.

**Conclusiones:**

La violencia contra mujeres embarazadas está presente en todo el país, afectando a todos los grupos de raza/color de piel, edades y niveles educativos. Sin embargo, se identificaron variaciones y especificidades entre las regiones. De este modo, los datos obtenidos representan una fuente relevante de información y pueden subsidiar la planificación e implementación de intervenciones.

## Resumo

**Introdução:**

A violência contra gestantes é um problema de saúde pública multifatorial que pode agravar a saúde materna e fetal, sendo relevante sua análise para compreensão do fenômeno.

**Objetivo:**

Descrever a distribuição espacial das taxas de notificações de violência contra gestantes brasileiras.

**Materiais e métodos:**

Estudo ecológico de corte transversal das taxas de notificação de violência contra gestantes residentes no Brasil ocorridas em 2023, obtidas por meio do Sistema de Informações de Agravo de Notificação, com dados extraídos em março de 2025. Foi realizada a distribuição espacial das taxas de notificação de violência contra gestantes segundo indicadores socioeconômicos (raça/cor; faixa etária; escolaridade). Para a análise espacial foram empregados o Índice de Moran Global e Local, considerando as regiões imediatas como unidades de análise.

**Resultados:**

A taxa de notificações de violência contra gestantes no Brasil foi de 7,82 por mil nascidos vivos (19.851 notificações). Os estados do Amazonas, Roraima, Acre, Mato Grosso do Sul e Paraná se sobressaíram com altas taxas. Destacou-se a ocorrência em mulheres da raça/cor da pele parda, amarela e indígena, bem como com idade inferior a 20 anos e baixa escolaridade.

**Discussão:**

O Brasil é marcado por disparidades regionais, que se refletem nos indicadores de violência contra gestantes.

**Conclusões:**

A violência contra gestantes está presente em todo o país, afetando todas as raças/cores, faixas etárias e níveis de escolaridade. Entretanto, foram identificadas variações e especificidades entre as regiões. Dessa forma, os dados obtidos representam uma fonte relevante de informação, podendo subsidiar o planejamento e implementação de intervenções.

## Introduction

Violence against women is recognized as one of the most frequent violations of human rights. In 2022, approximately 89,000 girls and women were intentionally murdered worldwide^1^, and thousands more continue to face some form of violence daily^2^.

Violence can be present at different stages of a woman's life, and pregnancy is no exception^3^. The physical and psychological changes that occur during pregnancy, associated with the stigma of power imbalance in relationships, affective, financial and dependency issues, make women more sensitive and vulnerable, increasing the chances of experiencing various types of violence, with often irreparable consequences^2^-^4^.

Among the various forms of violence to which a pregnant woman may be subjected, the following stand out: physical violence, characterized by assaults that cause bodily harm or affect her health; psychological violence, which involves emotional damage, threats, embarrassment, loss of self-esteem, and humiliation; and sexual violence, which forces the woman to have unwanted sexual relations^5^. There is also obstetric violence, which violates the rights of the pregnant woman, neglecting her wishes and emotions and exposing her to unnecessary or harmful interventions^6^.

Literature shows that violence against pregnant women occurs heterogeneously around the world, with prevalence rates varying significantly between continents. Global estimates indicate that approximately 25% of pregnant women have experienced some type of violence during pregnancy. Regarding physical violence, the highest prevalence rates are observed in Africa, at approximately 16.30%, while the lowest occur in Europe, around 2.10%. Despite these estimates, significant information gaps persist, especially in South American countries such as Argentina, Chile, Colombia, Venezuela, and Bolivia, as well as regions like Russia, Kazakhstan, and Australia, where epidemiological data are scarce or nonexistent^7^. Furthermore, femicide during the pregnancy-puerperal period presents worrying rates in various regions of the world, even being the leading cause of maternal death in the United States^3^.

In Brazil, around 50% of women experience intimate partner violence for the first time during pregnancy^8^. The occurrence of this phenomenon in the country also presents regional variations. In 2019, 14,606 reports of violence against pregnant women were registered, with the highest concentration in the Southeast (40.10%), Northeast (20.10%), and South regions (19.40%)^9^. It is noteworthy that the occurrence of violence during pregnancy is directly related to serious harm to women's health, such as hemorrhages, abortions, inadequate use of prenatal care, mental disorders, among others. This harm also affects the fetus, increasing the risk of prematurity, low birth weight, and perinatal mortality^3^,^10^.

It is noteworthy that the effects of violence on maternal and fetal health can be preventable, since pregnancy offers many opportunities for screening and intervention within the health system, such as during prenatal care^3^. In this context, considering the heterogeneity of violence during pregnancy across communities and regions, the lack of comprehensive spatial analyses at the national level, and its high potential to cause preventable harm, this study is justified by identifying and mapping the areas with the highest occurrence of violence against pregnant women, contributing to the understanding of territorial dynamics and promoting the objectivity of public prevention policies, according to priority areas. The objective of this study was to describe the spatial distribution of reported rates of violence against pregnant women in Brazil.

## Materials and Methods

This is a cross-sectional ecological study of the spatial distribution of reported rates of violence against pregnant women in Brazil in 2023, conducted using the Strengthening the Reporting of Observational Studies in Epidemiology tool (STROBE).

Brazil, located in South America, below the Equator, has 26 Brazilian states and the Federal District, 510 immediate regions, and 5,570 municipalities^11^. It is a country of continental dimensions, marked by profound regional socioeconomic disparities, which justifies the investigation of spatial patterns of violence.

The data regarding reports of violence against pregnant women were obtained from the Notifiable Diseases Information System (SINAN), available for public consultation through the file transfer platform – Tabwin. The live birth data, used as a proxy for the number of pregnant women, were obtained from the Live Birth Information System (SINASC). Both systems are available on the website of the Department of Informatics of the Unified Health System (DATASUS). Data collection took place in March 2025.

**Figure 1:**
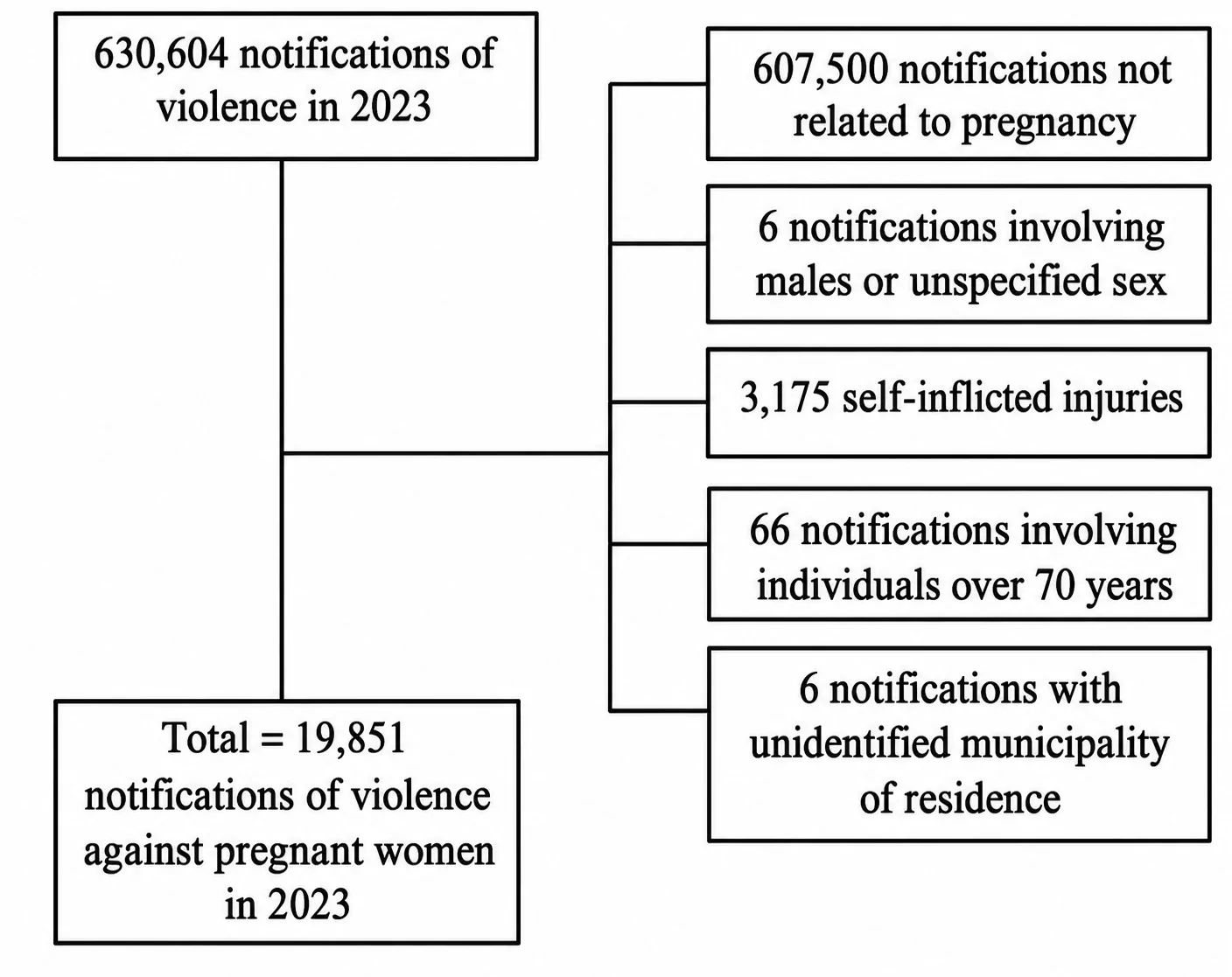
Exclusion criteria applied to the study.

After data collection, the notifications were filtered according to the mandatory notification form, including all types of interpersonal violence. Records of self-inflicted injury, male or unknown sex, and notifications of victims over 70 years of age were excluded, as these were considered outliers in this study because they represent exceptional cases that do not reflect the predominant profile of pregnant women. Cases where the immediate region was not identified were also excluded, since this information is essential for spatial analysis Figure 1. Data from 2023 were selected to obtain an updated sample for analysis, as it was the last year with data available in both systems at the time of data collection.

Cases of violence against pregnant women were analyzed considering variables related to the victim: age (<20 years, 20 to 34, and 35 and over, with younger women commonly presenting higher prevalences of violence)^12^; race/color (White, Black, Brown, Yellow and Indigenous, with the categories adopted in the study in accordance with the Brazilian Institute of Geography and Statistics (IBGE), Sinan and Sinasc); education (<8 years, 8 years or more, with the first group considered to be more vulnerable, as it is associated, according to the literature, with greater socioeconomic vulnerability)^13^.

The immediate regions were the units considered for the analysis of spatial distribution and autocorrelation. They group areas around urban centers, meeting the basic needs of the populations, such as employment, health and education^14^.

The rates were presented in choropleth maps using color scales, with lighter colors indicating lower rates and darker colors indicating higher rates. The maps were created using the cartographic base with the regional boundaries, available online as a shapefile (SHP) on the website of the Brazilian Institute of Geography and Statistics (IBGE). The rates of reported violence against pregnant women were calculated by dividing the number of reported cases of violence against pregnant women by the number of live births in the same location and period, multiplied by 1,000. In analyses stratified by race/color, age, and education level, the denominator corresponded to the number of live births in the same category.

Subsequently, the crude rates of reported violence against pregnant women were smoothed using the local empirical Bayesian rate, considering the spatial variability of the data and the influence of neighboring municipalities. Analyses were performed using exploratory spatial data techniques, employing spatial autocorrelation and visualization of rates in quintile and quartile intervals. The Global Moran's I index was used to assess the presence of spatial autocorrelation in the dataset, while the Local Moran's I index (Local Indicators of Spatial Association – LISA) was used to identify local patterns of spatial association and the formation of territorial clusters, allowing for the detection of clusters and the analysis of spatial variations between regions, classified as : High-High - immediate regions with high rates of violence against pregnant women. Surrounded by immediate regions that also have high rates; High-Low - immediate regions with high rates of violence against pregnant women, surrounded by immediate regions with low rates; Low-Low - immediate regions with low rates surrounded by immediate regions with low rates; Low-High - immediate regions with low rates that border immediate regions with high rates. The significance level adopted was p<0.05.

In accordance with CNS Resolution No. 466/2012, and since this research uses secondary data from a publicly available platform, posing no risk to the population or revealing the names of the subjects, it was not necessary to submit it to the Permanent Committee on Ethics and Research with Human Beings. However, it should be noted that the authors followed all the ethical precepts expected in conducting population studies.

Statistical analyses were performed using GeoDa software version 1.22, and the Quantum Geographic Information System (QGIS) software version 3.38.0 was used to create maps. All collected data are freely accessible and searchable in Mendeley Data^15^.

## Results

In 2023, 630,604 cases of violence were reported in Brazil, of which 3.15% were perpetrated against pregnant women. This study analyzed 19,851 reports of violence against pregnant women in Brazil, representing a rate of 7.82 reports per 1,000 live births. Nationally, the highest rates of violence against pregnant women were observed among self-declared Indigenous women (18.09 per 1,000 live births) and those under 20 years of age (26.85 per 1,000 live births). Regarding education, a high rate was observed in the category with missing information (277.43 per 1,000 live births), as well as those with less than 8 years of schooling (19.46 per 1,000 live births) Table 1.

**Table 1:** Distribution of reported cases of violence against pregnant women, according to race/ color, age, and education level. Brazil, 2023.

	Violence % (n)	Live Births % (n)	Rate per 1,000 LB
Brazil	100.00 (19851)	2 537,511	7.82
Race/color			
White	29.30 (5816)	840,041	6.92
Black	11.91 (2365)	196,323	12.05
Yellow	0.95 (189)	11,956	15.81
Brown	52.61 (10444)	1 413,597	7.39
Indigenous	2.70 (536)	29,622	18.09
Ignored	2.52 (501)	45,972	10.90
Age			
Under 20 years old	41.02 (8142)	303,266	26.85
20 to 34 years old	26.96 (9323)	1 778,936	5.24
35 or more	12.02 (2386)	455,278	5.24
Education			
8 years	28.46 (5649)	290,246	19.46
8 years or older	47.99 (9526)	2 230,168	4.27
Ignored	23.56 (4676)	16,855	277.43

LB = live births; n = absolute number of cases; % = percentage

**Figure 2:**
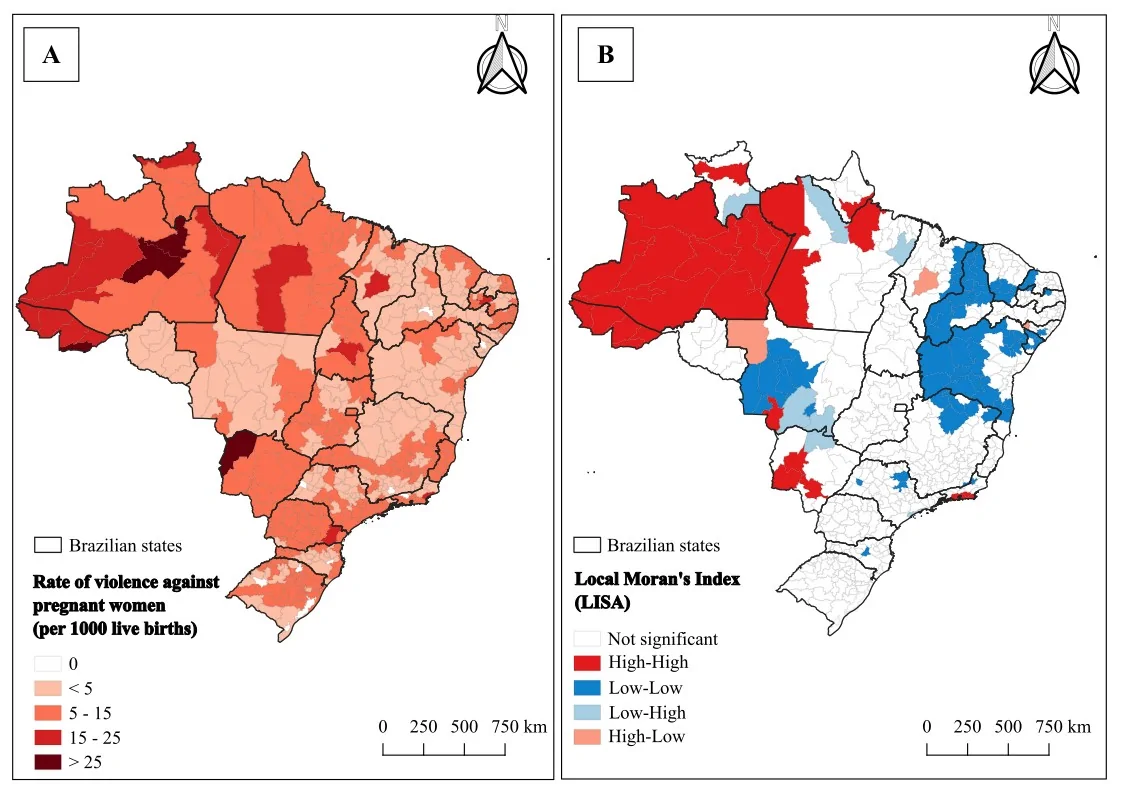
Distribution of reported rates of violence against pregnant women and their clusters, Brazil 2023.

In the spatial analysis, the results obtained through the Global Moran's I indicated a positive spatial autocorrelation of rates of violence against pregnant women, with Moran's I = 0.374 and z-score = 14,5871, evidencing a non-random spatial pattern. The spatial distribution also showed that the highest rates are heterogeneously spread across the country. Among the federative units, it is noted that the states in the North Region, especially Acre, Amazonas, and Roraima, have the highest occurrence of violence against pregnant women. However, the states of Mato Grosso do Sul and Paraná also showed high rates. In contrast, states such as Rondônia, Sergipe, Piauí, Bahia, and Rio Grande do Sul showed a predominance of regions with lower or zero rates Figure 2A.

In the immediate regions, Corumbá-MS, Manacapuru-AM, Coari-AM, and Brasiléia-AC stand out with high rates (53.52; 27.88; 26.25 and 25.76 per 1,000 live births, respectively). The lowest rates corresponded to the immediate regions of Simplício-PI, Caxambu-MG, Dracena-SP, Ituporanga-SC, Santiago-RS, Tapejara-RS, Sananduva-RS, Itabaiana-SE, and Valença-BA, with a zero rate Figure 2A.

The analysis of spatial autocorrelation revealed immediate regions that formed significant spatial clusters. 29 immediate regions with direct or positive spatial correlation were identified, classified as high-high clusters (5.69% of the total immediate regions in Brazil). Indicating immediate regions with a high rate of reported violence against pregnant women, neighboring other equally high regions. These clusters can be visualized on the map by the dark red areas, corresponding to six distinct clusters, the most significant of which is in the Northern Region of the country, encompassing the states of Acre, Amazonas, and Pará Figure 2B.

On the other hand, 70 immediate regions formed 8 clusters with classification. Low-low (13.73% of the total immediate regions), that is, immediate regions with a low rate of reported violence against pregnant women surrounded by others that also have low rates. These regions were shown in dark blue, with the largest group encompassing Maranhão, Piauí, Bahia, Ceará, Rio Grande do Norte, Sergipe, Alagoas, and northern Minas Gerais Figure 2B.

Furthermore, patterns of negative or inverse spatial correlation are observed, characterized by contrasts between local values and those of neighboring regions. Three immediate regions presented the high-low pattern (0.59%), and seven were classified as low-high (1.37%) Figure 2B.

**Figure 3:**
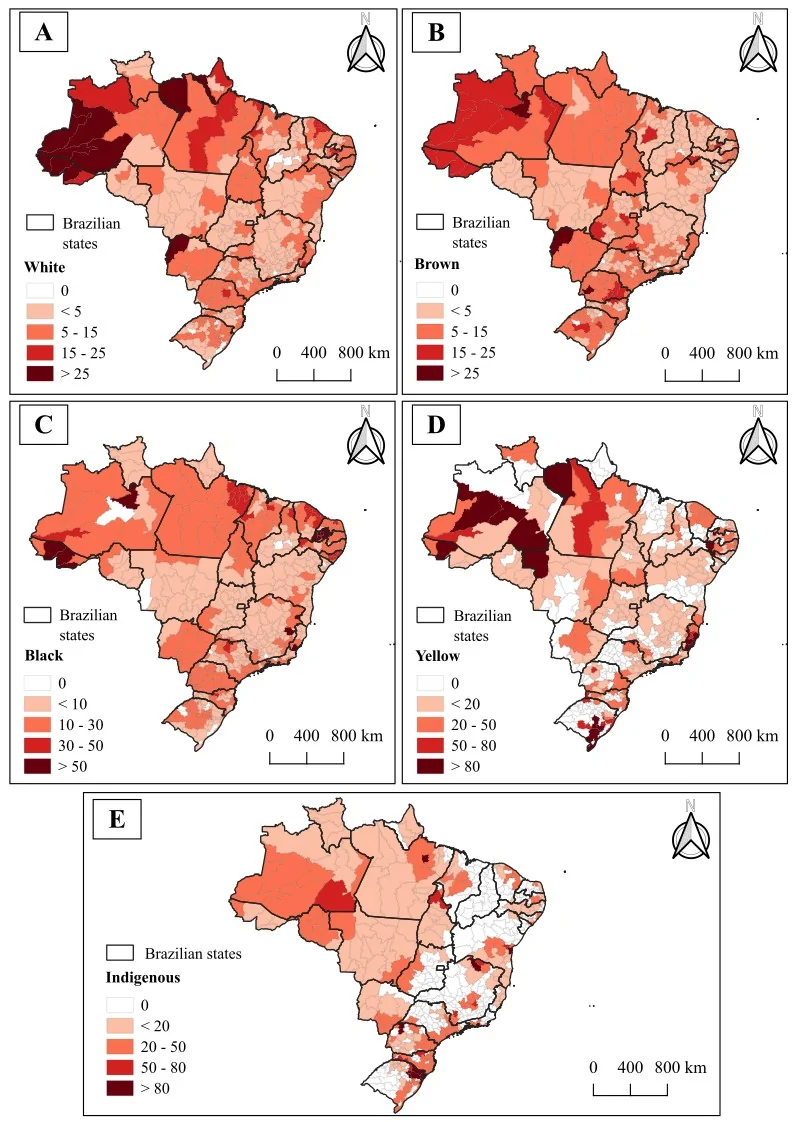
Spatial distribution of violence against pregnant women in Brazil, according to the victim's race/color. Brazil, 2023.

Regarding the distribution by race/color, it was found that reports of violence against pregnant women occur with distinct patterns among the different groups. The occurrence among brown-skinned pregnant women stood out for its scope, widely dispersed across various regions Figure 3B. While the occurrence among yellow and indigenous races/colors stands out for its magnitude, presenting rates higher than 80 reports per 1,000 live births Figure 3D and 3E.

It is evident that violence against pregnant women of Asian descent occurs predominantly in the North of the country, especially in the states of Amazonas, Acre, and Pará. However, rates exceeding 80 are also found in other regions (Northeast, Central-West, and South) Figure 3D. Similarly, rates among Indigenous people generally predominate in the North of Brazil, extending to the western portion of the Central-West (Mato Grosso and Mato Grosso do Sul) and the Southern states Figure 3E.

*(A) White. (B) Brown. (C) Black. (D) Yellow. (E) Indigenous.*

**Figure 4:**
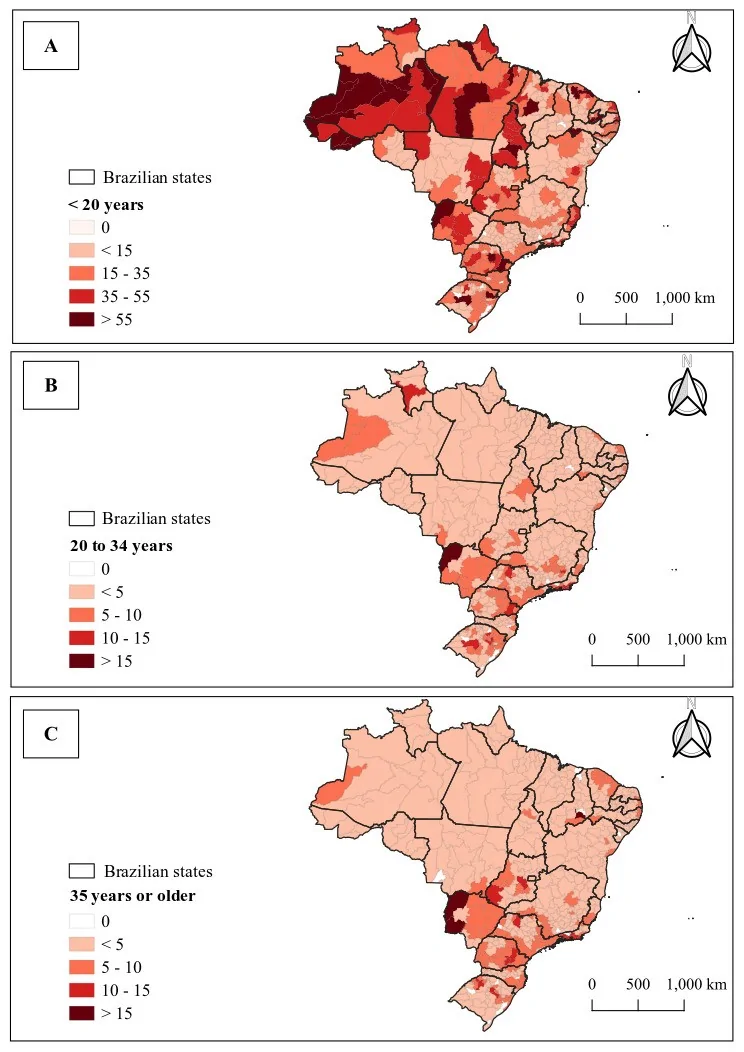
Spatial distribution of violence against pregnant women in Brazil, according to the victim's age group. Brazil 2023.

Regarding the distribution of violence against pregnant women according to age group Figure 4, a significant disparity in violence rates is evident across the different age groups studied. Young women under 20 years of age were the most affected, especially in the North and Central-West regions of the country, particularly in the states of Amazonas, Acre, Amapá, Rondônia, and Mato Grosso do Sul Figure 4A. Among pregnant women aged 35 or older, the rates were the lowest in terms of intensity, with a predominance of rates below 5 notifications, except for some immediate regions in the states of Mato Grosso do Sul, Paraná, and Rio Grande do Sul, where rates above 10 were identified Figure 4C.

**Figure 5:**
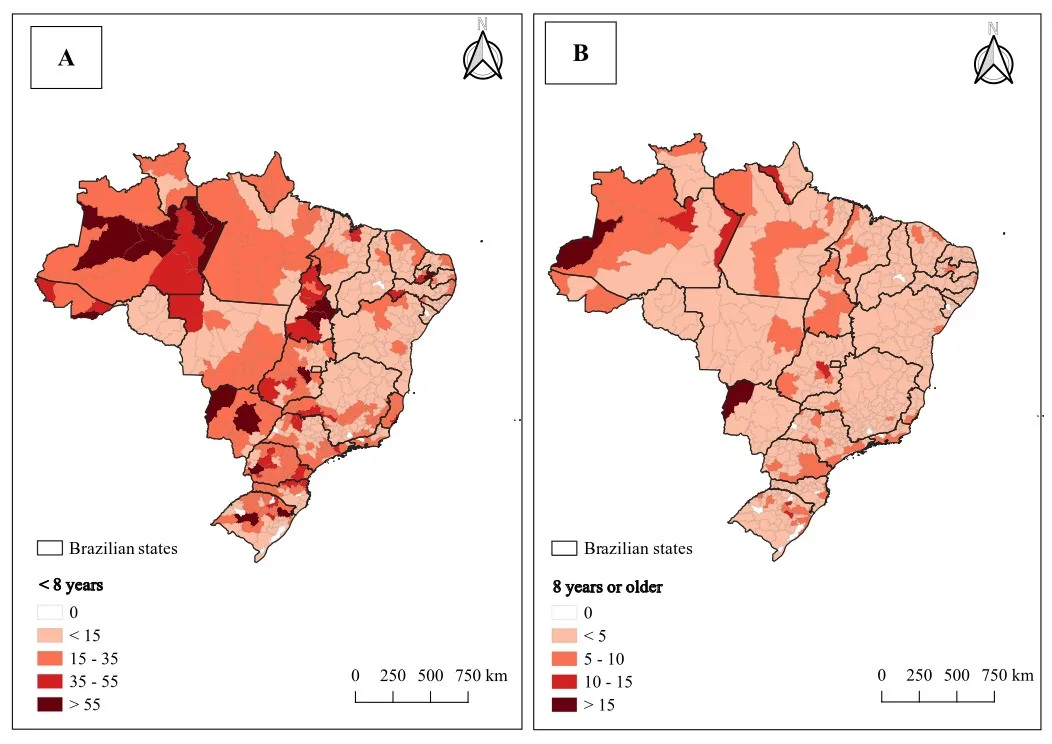
Spatial distribution of violence against pregnant women in Brazil, according to the victim's education level. Brazil, 2023.

Regarding the education level of pregnant women, it is evident that women with less than 8 years of schooling were the main victims, reaching rates exceeding 55 notifications per 1,000 live births in some immediate regions, mainly in the North and Central-West regions of the country Figure 5A. However, it is observed that violence also affects pregnant women with 8 or more years of schooling, although in most cases, the rates remain below 5 notifications per 1,000 live births. The main exceptions occur in localized hotspots in the states of Amazonas, Pará, Tocantins, and Mato Grosso do Sul, where immediate regions with punctual increases were identified Figure 5B. However, it should be noted that there were notifications in which the field was ignored or not filled in (23.56%).

## Discussion

This study sought to identify the spatial distribution of rates of violence against pregnant women in Brazil in 2023. During the research, no studies were found that explored this topic from a spatial perspective, making this one of the first at the national level. This allows for important contributions, such as identifying areas with a higher concentration of cases, helping to understand the territorial dynamics of violence against pregnant women and to highlight potentially more vulnerable regions.

The national rate was 7.82 cases per 1,000 live births, indicating a worrying scenario, especially because it involves the so-called double vulnerability of the woman and the fetus, which highlights the need for specific and qualified care from health services. This context demands sensitive support and comprehensive interventions from the care networks, focusing on the protection of the pregnant woman and the guarantee of her rights^4^.

Additionally, the study conducted by Soares and colleagues in 2023 characterized the main causes of death among women with reported interpersonal violence during pregnancy, observing that in the period from 2011 to 2017, 45.20% of pregnant women with reported violence during pregnancy died. This observation is concerning and amplifies the need for immediate interventions to prevent violence and consequently femicide during pregnancy^3^.

Beyond the Brazilian context, violence against pregnant women is not an isolated phenomenon, being widely recognized in different international scenarios. In the Latin American context, a worrying situation is observed, as in Peru, where the prevalence of violence against pregnant women reached 29.44%^16^. In high-income countries, such as the United States of America, evidence indicates that homicides occurring during pregnancy or in the 42 days following its termination more than doubled the main traditional causes of maternal mortality^17^.

In this study, using the spatial scanning method, it was possible to identify the area’s most vulnerable to violence against pregnant women, with high-high clusters concentrated predominantly in the North region of the country, particularly in the states of Acre, Amazonas, and Pará. Previous studies had already indicated this region as one of those with the fastest-growing rates of violence in Brazil^18^. Furthermore, data show that Amazonas and Acre register the highest proportions of women who reported having suffered or witnessed domestic violence (57% and 54%, respectively), with rates higher than the national average, which is 48%^19^.

The concentration of these cases in this region may suggest geographical peculiarities. Geographical obstacles hinder access to support services and the perception of the severity of the cases^20^. Rural women are more prone to violence, since they require greater dependence on their partner and do not know who or what to turn to, facing difficulties in asking for help^21^.

Furthermore, Brazil is marked by profound regional disparities, which are reflected in social, economic and health indicators. The North and Northeast regions concentrate the worst results in these indicators, evidencing a scenario of historical and structural inequality in relation to the other regions of the country^22^,^23^.

Although the states of Rondônia, Sergipe, Piauí, and Bahia present lower rates of violence against pregnant women, it is worth highlighting that, according to the Atlas of Violence in Brazil (2025), these states have high rates of female homicide. According to this document, Bahia is considered the most violent state for women, and the states of Rondônia and Piauí have the highest incidence of femicide in Brazil in the years 2018 to 2023. As for the states of Mato Grosso do Sul and Paraná, which presented high rates of violence against pregnant women in this study, Mato Grosso do Sul appears as a state with a low proportion of femicide in the Atlas of Violence, even showing a decrease in the period from 2018 to 2023, and Paraná follows the proportion presented for Bahia^24^.

The discrepancies between the data on violence against pregnant women and femicide may indicate difficulties on the part of some states in reporting the data, since it is expected that regions more vulnerable to femicide are also vulnerable to violence against women, including pregnant women.

Regarding the race/color variable, the data reveal a predominance of cases among brown women, as well as a higher magnitude of rates among those of yellow and indigenous race/color. This finding corroborates evidence that black, brown, and ethnic minority women are the main victims of violence^25^.

Furthermore, IBGE data reveals profound inequalities in access to work and education. Approximately 69% of managerial positions are held by white people, demonstrating the underrepresentation of racialized groups in leadership positions^26^. In education, the illiteracy rate among mixed-race people is 8.8%, more than double that observed among white people (4.3%). Among the indigenous population, this rate is even more alarming, reaching 16.1%^27^.

The high rates observed among self-declared Asian pregnant women, especially in the Northern Region, should be interpreted with caution due to the instability effect of rates in small populations. In this region, which is home to only 0.20% of the country's Asian population, a small absolute number of cases can result in very high proportional rates^28^.

Regarding indigenous women, it is observed that, despite the transformations that have occurred since the colonial period, many are still victims of sexual exploitation. Stereotypes persist that reduce them to hypersexualization and fetishization, perpetuating violence. In addition, contact with white men and the introduction of alcohol and other drugs into indigenous communities have intensified the violence perpetrated by indigenous men against their partners and other women in the group^29^.

The prevalence of violence against pregnant women of Asian and Indigenous descent in the Northern Region, especially in Amazonas, Acre, and Pará, is also linked to the phenomenon of plant or mineral extraction. In these areas, one observes the so-called "corrutelas," support points that operate as kitchens, bars, supply stores, lodging, leisure, and socialization spaces. In these "corrutelas," prostitution and sexual exploitation of girls and women are common, including alcohol and drug abuse, where gender is a fundamental marker. There are reports of assaults and even murders when the men's wishes are thwarted^24^.

Regarding age range, violence was observed in all cycles and phases of a woman's life, indicating that it is a phenomenon that transcends the age^25^. However, the highest rates were among those under 20 years of age, possibly due to socioeconomic and emotional dependence. This dependence, frequently associated with low education and lack of insertion in the labor market, contributes to the vulnerability of young women^30^. In this context, violence can emerge as an expression of structural limitations and, in some cases, be naturalized or tolerated as a survival strategy.

Among young women in this age group, jealousy on the part of intimate partners remains a relevant factor. Controlling behaviors are often accepted and interpreted as demonstrations of care, contributing to the trivialization and invisibility of abusive dynamics^31^. On the other hand, it is also observed that increased access to information and public services, such as security, education, and health, has the potential to strengthen strategies for breaking the cycle of violence and encouraging the reporting of aggressors, representing progress in confronting these situations of oppression. In this context, the role of epidemiological surveillance stands out, acting decisively in identifying a pregnant woman aged 14 or younger, carrying out the mandatory notification of rape of a vulnerable person, as provided for in current Brazilian legislation, which may result in a greater number of notifications in the under-20 age group^25^,^32^.

Among pregnant women of advanced age, the numbers of perpetrated violence tend to be lower, which may indicate greater family and personal life planning. Many women in this age group generally have greater independence, emotional stability, better access to contraceptive methods, and a desire to prioritize their professional careers to achieve financial stability before starting a family^33^. Furthermore, this greater preparation for pregnancy results in greater assertiveness in choosing a partner and more stable relationships, contributing to a better experience during the postpartum period and less exposure to violence.

Regarding the educational level of the victims, previous studies corroborate that there is a higher prevalence of violence cases among pregnant women with low levels of education, highlighting mainly that the majority of women in situations of violence had less than 8 years of schooling^13^, suggesting an inverse correlation between the level of education and tolerance of violence; the greater the access to education, the lower the acceptance of abusive situations. Furthermore, the literacy deficit constitutes a factor that makes the pregnant woman financially dependent on others, since education is related to the expansion of opportunities in the job market and, consequently, to better living conditions^34^.

This study has some limitations. One of them is the use of secondary data, which is subject to incomplete information, the possibility of underestimation due to underreporting of cases, and the impossibility of establishing direct causal inferences. However, the data show promise as they are original information, offering valuable and essential insights for understanding the phenomenon under study, and contributing to guiding future research.

## Conclusions

Violence against pregnant women is present throughout the country, highlighting its multifactorial and complex nature, affecting women of different races/colors, age groups, and educational levels. However, regional variations were identified, with higher rates in the states of Amazonas, Roraima, Acre, Mato Grosso do Sul, and Paraná, indicating significant disparities between regions. Greater vulnerability was observed among pregnant women under 20 years of age, those who self-identified as mixed-race, Asian, or Indigenous, and those with low levels of education, indicating greater exposure of these groups to violence.

It is important to highlight that the ethno-racial characteristics of violence against pregnant women can be imprecise. Notifications of violence against Indigenous and Asian women, for example, tend to be lost in the "mixed-race" category, since they are not always based on self-declaration. In other words, it is possible that violence against Indigenous and Asian pregnant women is underreported in some areas and is even more representative than observed in this study.

Protective measures, information, and support for victims are essential to ensure that this cycle is broken. It is also necessary to implement more effective public policies, especially strengthening primary healthcare, where prenatal care is the gateway for pregnant women into the public health system and allows for the creation of bonds, opening space for debate and actively listening to women, identifying and addressing their vulnerabilities.
